# Proteomics Reveals Distinct Changes Associated with Increased Gamma Radiation Resistance in the Black Yeast *Exophiala dermatitidis*

**DOI:** 10.3390/genes11101128

**Published:** 2020-09-25

**Authors:** Zachary S. Schultzhaus, Janna N. Schultzhaus, Jillian Romsdahl, Amy Chen, W. Judson Hervey IV, Dagmar H. Leary, Zheng Wang

**Affiliations:** 1 Center for Bio/Molecular Science & Engineering, Naval Research Laboratory, Washington, DC 20375, USA; zachary.schultzhaus@nrl.navy.mil (Z.S.S.); janna.schultzhaus.ctr@nrl.navy.mil (J.N.S.); judson.hervey@nrl.navy.mil (W.J.H.IV); dasha.leary@nrl.navy.mil (D.H.L.); 2 National Research Council, Postdoctoral Fellowship Program, US Naval Research Laboratory, Washington, DC 20744, USA; jillian.romsdahl.ctr@nrl.navy.mil; 3 Virginia Tech Carilion School of Medicine, Roanoke, VA 24016, USA; ajchen@vt.edu

**Keywords:** black yeast, melanin, proteomics, radiobiology, translation

## Abstract

The yeast *Exophiala dermatitidis* exhibits high resistance to γ-radiation in comparison to many other fungi. Several aspects of this phenotype have been characterized, including its dependence on homologous recombination for the repair of radiation-induced DNA damage, and the transcriptomic response invoked by acute γ-radiation exposure in this organism. However, these findings have yet to identify unique γ-radiation exposure survival strategies—many genes that are induced by γ-radiation exposure do not appear to be important for recovery, and the homologous recombination machinery of this organism is not unique compared to more sensitive species. To identify features associated with γ-radiation resistance, here we characterized the proteomes of two *E. dermatitidis* strains—the wild type and a hyper-resistant strain developed through adaptive laboratory evolution—before and after γ-radiation exposure. The results demonstrate that protein intensities do not change substantially in response to this stress. Rather, the increased resistance exhibited by the evolved strain may be due in part to increased basal levels of single-stranded binding proteins and a large increase in ribosomal content, possibly allowing for a more robust, induced response during recovery. This experiment provides evidence enabling us to focus on DNA replication, protein production, and ribosome levels for further studies into the mechanism of γ-radiation resistance in *E. dermatitidis* and other fungi.

## 1. Introduction

The biological effects of γ-radiation exposure are well known. Some damage occurs when ionizing sources directly contact cellular components, yet most of the damage occurs when photons interact with water molecules throughout the cell, resulting in the radiolytic production of various reactive oxygen species. These products, such as hydrogen peroxide, superoxide, and hydroxyl radicals [1], rapidly react with and damage nearby biomacromolecules, particularly DNA and proteins. The resulting damage to the DNA, primarily in the form of double strand-breaks (DSBs), will lead to cell death if left unrepaired [2]. In general, the repair of DSBs is also well characterized—it occurs either through homologous recombination (if homologous chromosomes or sister chromatids are present) or non-homologous end-joining [3,4]. Cell viability also depends on whether the proteins involved in these processes are able to perform their functions after withstanding the damage caused by the products of radiolysis [5].

The effects of γ-radiation exposure and the mechanisms involved in DNA repair are relatively widespread and predictable throughout eukaryotes [4,6]. However, γ-radiation resistance can vary widely based on genetic background, developmental stage, and the environment [7,8,9,10,11], suggesting that the possibility of survival after exposure can be adjusted based on either an organism’s response or its physiological state prior to irradiation. This hypothesis suggests that radiation-induced damage may possibly be prevented, which is still a largely unmet goal in the field of radiological and nuclear countermeasures. However, to begin to understand the conditions and genetic variations that assist in producing γ-radiation resistance requires further analysis of organisms that vary in susceptibility.

With this in mind, we are currently studying the radiobiology of the melanized yeast *E. dermatitidis*. *E. dermatitidis* (Figure S1) has long been used as a model for a group of more than 100 constitutively melanized yeasts (a.k.a. dermatophytes) that inhabit a variety of extreme and human-associated environments, and are of interest for their pathogenicity, their stress tolerance (e.g., for extreme temperatures, desiccation, and ionizing radiation (IR)), and their potential for bioremediation [12,13,14,15,16,17]. Its use as a model for this group of organisms stems from its importance in the clinic as a neurotrophic pathogen presenting often as an external skin disease but occasionally causing an invasive disease that is almost invariably fatal. However, equally important are other features that make *E. dermatitidis* amenable for experimentation, including: A modest genome size among dermatophytes; a relatively quick growth rate (reaching stationary phase in < 48 h); a relatively high transformation and homologous recombination rate, which has allowed for functional characterization of dozens of its genes. Other major areas of investigation with *E. dermatitidis* have been its resistance to γ-radiation-induced damage [9,18,19,20,21] and its melanin biosynthetic pathway, which is constitutively active in *E. dermatitidis* and has been scrutinized at the phenotypic (e.g., virulence) and genetic level for more than twenty years [22,23].

Previously, we have made several advancements that situate *E. dermatitidis* as an attractive model for γ-radiation research. First, we characterized the growth kinetics and transcriptomic response during exposure to relatively low but chronic γ-radiation. In that publication, we observed that an induction of the oxidative stress response, ribosome production, and carotenoid biosynthesis were associated with this condition [15]. Second, we obtained a high quality sequence of the genome, opening up the opportunity to perform functional genomic and proteomic analyses of this organism’s radiation response [24]. Third, we performed transcriptomics on *E. dermatitidis* cells recovering from a single, high dose of γ-radiation. In this case, there was no noticeable change in the transcription of oxidative stress proteins. Rather, DNA damage transcripts were greatly increased in expression, while ribosomes and other growth-associated genes were strongly repressed, highlighting the predominance of DNA repair and cell cycle arrest during recovery [9]. Most recently, we have developed, through adaptive laboratory evolution, several strains of *E. dermatitidis* that possess increased γ-radiation resistance, in order to allow for a direct comparison between closely related strains that differ in this phenotype.

A detailed study of each of these “evolved” strains, including their genome sequences and differential transcriptomic responses to γ-radiation exposure, will be published in a separate manuscript. However, changes in transcript abundance do not always provide good evidence of which proteins are important for γ-radiation resistance [9,25]. To take advantage further of these strains, we performed comparative, shotgun proteomics on wild type (WT) and one of the most highly-resistant evolved strains prior to and after exposure to a moderate dose of γ-radiation. In achieving this, we accomplished several goals, including: Developing a rapid method for efficient protein extraction from *E. dermatitidis* cells that avoids hazardous chemicals and laborious cell lysis techniques, determining that few proteins can be seen changing in their observed amounts within the first hour after γ-radiation exposure, and identifying two sets of proteins—single-strand DNA binding proteins and particularly ribosomal proteins, that are unique markers of the evolved strain proteome. These findings suggest at least two pathways to focus on for understanding the increased resistance of this strain and situate us to carry out more proteomic experiments targeting the radiobiology of *E. dermatitidis* in the future.

## 2. Materials and Methods

### 2.1. Development and Growth of Fungal Strains Used E. dermatitidis

The *E. dermatitidis* WT (Ed8656) strain has been used previously and is the strain from which the reference genome is derived [23,24]. The non-melanized mutant (Wd*PKS1*) pictured in Figure S1 was developed from WT in a prior reference using standard gene disruption techniques [19,20]. The Evolved strain (a.k.a. WT.15.2.2) was obtained in the following manner: For four biological replicates, a single colony of WT *E. dermatitidis* each was inoculated into 2.5 mL of liquid Yeast Peptone Dextrose (YPD) medium and grown for three days at 30 °C and 225 RPM, after which 100 µL was removed and exposed to 4500 Gy of γ-radiation from a Cobalt-60 source, and re-inoculated into 2.4 mL of liquid YPD and grown for three days again. The irradiation and sub-culturing was repeated 15 times, with a dose of 4500 Gy at time point 1–5, 5000 Gy for 6–10, and 5500 Gy for rounds 11–15. After the fifteenth round, irradiated cultures were diluted, plated, and two of the fastest-recovering single colonies were purified for each biological replicate for further characterization. This resulted in eight strains derived from WT that exhibited increased resistance to γ-radiation exposure of varying degrees. One of these strains, named WT 15.2.2 was used in this current study, while and more detailed information about its characteristics and those of the other strains will be published elsewhere.

To obtain samples that allowed for comparison of lysis methods, three WT cell cultures were started in 2 mL of liquid Yeast Peptone Dextrose (YPD) medium from individual single colonies grown on solid YPD agar plates. These cultures were grown for 48 h at 30 °C and 200 RPM, at which time cultures were diluted 1:1000 in 2.5 mL of liquid YPD medium and incubated an additional 48 h at 30 °C and 200 RPM. At this point, each culture was split into 2 × 1 mL aliquots, pelleted through brief centrifugation (5000× *g*, 1 m), and then flash frozen (after removing the supernatant) and stored at −80 °C until processing occurred.

### 2.2. Collection and Analysis of Genome Sequence of Evolved Strain

Genomic DNA for whole genome sequencing was obtained by inoculating cells from a single colony into 3 mL of liquid YPD, which were grown at 30 °C while shaking at 225 RPM for 3 days. DNA was extracted using the OmniPrep for Yeast kit (G-Biosciences, St. Louis, MO, USA). Library preparation and NovaSeq Illumina paired-end sequencing were conducted at the Yale Center for Genome Analysis. Raw WGS reads are available in NCBI SRA under accession number PRJNA635404.

Reference genome and annotation files for Ed8656 were downloaded from the EnsemblFungi web portal (https://fungi.ensembl.org/). The 150 base read Illumina sequences were trimmed using Trimmomatic v. 0.36 [26] and quality was checked using FastQC v 0.11.7 [27]. Reads were mapped to the reference genome using the Burrows-Wheeler Aligner (BWA) software package [28] and sorted BAM files were generated using SAMtools v 1.9 [29]. Picard Tools’ MarkDuplicates (https://broadinstitute.github.io/picard/) was used to remove PCR artifacts and variants were called using GATK v 3.8.7 [30]. Specifically, reads containing putative INDELs were realigned using GATK’s IndelRealigner, variants were called using GATK’s Haplotype Caller, Variant Call Format (VCF) files were combined using GATK’s Genotype GVCFs, and VCF files were filtered using GATK’s Variant Filtration based on the following cutoffs: (SNPs: QD  <  2.0, MQ  <  40.0, QUAL  <  100, FS  >  60.0, MQRankSum  <  −12.5, SOR  >  4.0, ReadPosRankSum  <  −8.0; INDELs: QD  <  2.0, FS  >  200.0, MQRankSum  <  −12.5, SOR  >  4, InbreedingCoeff  <  −0.8, ReadPosRankSum  <  −20.0). The resulting high-quality variants were annotated and functional effects were predicted using SnpEff [31].

### 2.3. Irradiation of E. dermatitidis

To obtain irradiated samples for proteomic analysis, both the WT and the Evolved strains were prepared in the following manner: Six cultures for each strain, representing six biological replicates, were initiated through the inoculation of 2 mL of liquid YPD medium with approximately 5 µL of cells from a single colony of the appropriate strain growing on a plate of YPD agar. These cultures were incubated for 48 h at 30 °C and 200 RPM, at which time cultures were diluted 1:1000 in 10 mL of liquid YPD medium and incubated an additional 72 h at 30 °C and 200 RPM such that cells grew entirely as yeast and reached stationary phase. An aliquot was then removed for cell counting using a Cellometer. Cultures were then spun down at 4000 G for 5 m at room temperature and resuspended to 5 × 10^8^ cell/mL in ultrapure H_2_O. From this concentrated solution, 500 µL was removed for each of six replicates for both control and irradiated conditions (12 samples, 6 mL total). Experimental samples were exposed to a dose of 500 Gy of γ-radiation from a Cobalt-60 source producing approximately 250 Gy/min. Samples were placed on ice prior to and after irradiation until recovery was initiated. For recovery, 500 µL of 2× concentrated YPD was added to each cell culture, and the cultures were allowed to incubate for 1 h at 30 °C and 200 RPM in 15 mL cell culture tubes. This is not enough to result in an increase in cell number for *E. dermatitidis* [15,22], but has been determined previously by our group to be enough time to allow for a robust response to irradiation [9]. After the incubation period, a small aliquot (10 µL) was removed from each sample and diluted to an appropriately low concentration before plating to determine survival. The rest of the cells were then transferred to microcentrifuge tubes, pelleted through brief centrifugation (5000× *g*, 1 m), and after removing the supernatant, the tubes were flash frozen in liquid nitrogen and stored at −80 °C until processing for protein extraction.

In addition, to determine characteristics of the survival curve for both the WT and Evolved strains, we cultured cells in a similar manner as above. However, prior to irradiation we adjusted them to 1 × 10^8^ cells/mL in ultrapure H_2_O and removed two aliquots of 100 µL of this suspension for each dose. Aliquots were kept on ice prior to and after irradiation, and after irradiation, cells were diluted, again in ultrapure H_2_O, to an appropriate concentration that would allow for counting of surviving colonies. Two YPD plates were prepared counted for each replicate, resulting in four plates per dose overall. After five days of incubation at 30 °C colonies were counted and survival was determined by adjusting for dilution and comparing with colony formation on plates with non-irradiated cells.

### 2.4. Cell Lysis and Protein Extraction

For protein extraction, cell pellets were removed from storage at −80 °C and immediately resuspended in 1 mL of 100 mM ammonium bicarbonate + 10% n-propanol. For bead-beating, this 1 mL sample was added to a sterile 2 mL Bead Ruptor tube containing 0.5 mm glass beads (VWR, Radnor, PA, USA) and bead-beating proceeded using a Cole-Parmer mini bead beater (Vernon Hills, IL, USA) at max speed, processing each sample twice at maximum speed for one minute with a one minute incubation on ice in between. For the OneShot treatment, cell pellets were resuspended in 5 mL in the same buffer, and resuspended cells were passed through the OneShot (Pressure Biosciences Inc., Easton, MA, USA) at the maximum pressure settings (40 kPa) twice. After bead-beating and OneShot processing, aliquots of lysate were removed for protein concentration quantification using the Pierce BCA Protein Assay Kit (Thermo Fisher Scientific, Waltham, MA, USA), and the remaining lysate was flash frozen in liquid nitrogen.

### 2.5. Proteolysis

Proteolysis was performed using pressure cycling technology (PCT) where samples are exposed to bursts of high levels of hydrostatic pressure. PCT facilitates protein unfolding and enzymatic access to cleavage sites, reducing the time needed for digestion [32,33,34,35,36]. Lysate containing ten micrograms of protein was added to a tube designed for use in the Barocycler (Pressure Biosciences Inc., Easton, MA, USA) with a 1:50 ratio of sequencing grade modified trypsin (Promega, Madison, WI, USA) as previously described [32,37,38]. The volume was adjusted to 150 µL 100 mM ammonium bicarbonate + 10% n-propanol. Digestion occurred under pressure (90 cycles of 45 kpsi, 50 s on at 10 s off, 50 °C). Digestion was stopped by adjusting pH with formic acid, and samples were dried until complete desiccation in a SpeedVac.

### 2.6. Liquid Chromatography and Tandem Mass Spectrometry

100 ng of the sample was injected into LC-MS/MS system for proteomics analysis using U3000 LC coupled to Orbitrap Fusion Lumos mass spectrometer (Thermo Scientific, Waltham, MA, USA). The autosampler loaded sample onto trap column (PepMap 100, C18, 300 um ID × 5 mm, 5 um, 100 A) via the loading pump at a 5 uL/min flowrate of 98% solvent A (0.1% formic acid in water) and 2% solvent B (0.1% formic acid in acetonitrile) for three minutes. The analytical pump eluted peptides at 300 nL/min from the trap onto the analytical column (Acclaim PepMap RSLC, 75 µm ID × 150 mm, C18, 2 µm, 100 A) using a two-step gradient of increasing solvent B (18% over the first 80 min, followed by a 60% increase over 15 min). Mass spectra were acquired on a Fusion Lumos Orbitrap equipped with a Nanospray Flex Ion Source in data-dependent acquisition mode with a 3 s cycle times. A survey scan range of 400–1600 Da was acquired on the Orbitrap detector (resolution 120 K). Maximum injection time was 50 ms and AGC target was 400,000. The most intense ions with charges of 2–7 were fragmented using HCD (higher-energy collisional dissociation), and ions were excluded for 30 s from subsequent MS/MS submission.The IonTrap detected MS/MS with a 35 ms injection time and an AGC target of 10,000. Upon Publication Release request approval, all raw data collected for this study will be uploaded to the ProteomeXchange Consortium via the PRIDE partner repository.

### 2.7. Data Analysis

Few mutations were observed between the two strains, and the vast majority of those that did were not expected to change the amino acid sequences of any proteins (for example, frameshift mutations such as indels likely result in misfolded proteins that are degraded and non-functional), so all raw files were searched against the UniProt reference predicted proteome of *E. dermatitidis* using MaxQuant v1.6.10.43 (http://www.maxquant.org). Default settings were maintained with the following deviations: Variable modifications included oxidation (M) and acetyl (Protein N-term), and the label-free quantification and match between runs features were enabled. The proteinsgroups.txt file containing LFQ intensities based on the MS level peak areas was loaded into R and analyzed for differential expression with the DEP package [39]. Data were filtered to exclude proteins with excessive missing values (Lysis dataset-only proteins with no more than one missing value was detected in either condition were allowed; Evolution/Radiation dataset—no missing values greater than 2 in any replicate were allowed). Missing values were then imputed using a mixed imputation method with missing not at random proteins (defined when proteins were missing from all replicates in at least one condition) imputed using a zero method (all NA cells replaced with 0 values), and missing at random proteins were imputed using the MinDet (minimum detection) algorithm. The statistical cutoff for significance was set at α = 0.05 and log_2_ fold change of 1.0. All plots were generated with the DEP or VennDiagram package [40].

Analysis of the proteins that were changed in relative amounts for each strain and condition was performed to identify enriched gene sets in the following manner (and as described for transcriptomics previously [9,41]): Using FungiFun2 (https://sbi.hki-jena.de/fungifun/fungifun.php [42,43]), the relevant lists of gene names were entered and searched for Gene Ontology—Biological Process categories that were significantly enriched using a Fisher’s exact test and an FDR cutoff of <0.01. This provided us with proteins that were overrepresented in the lists of those that were significantly changed in intensity or relative amount under certain strains or conditions.

## 3. Results

### 3.1. Radiation Resistance of Wild Type and Evolved E. dermatitidis Strains

**Figure 1:**
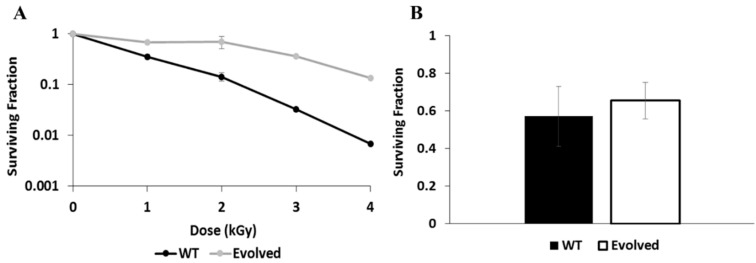
Resistance of wild type (WT) and Evolved strains to γ-radiation. (**A**) Survival curve showing the percent of cells recovered for each strain after exposure to increasing doses, up to 4 kGy. (**B**) Survival of each strain after exposure to a dose of 500 Gy and recovery in fresh medium for 1 h, measured from a subset taken from the same samples that were processed for proteomics.

The purpose of this study was to document the proteomic changes that occur in *E. dermatitidis* that are either in response to γ-radiation exposure, or are associated with greater γ-radiation resistance. Therefore, we first chose two strains to work with: The wild type strain (WT), which is moderately resistant to γ-radiation compared to other fungi [9], and a strain that we developed through adaptive laboratory evolution to exhibit increased γ-radiation resistance (referred to as Evolved or WTE, see Materials and Methods). As demonstrated in the survival curve in Figure 1A, the evolved strain was substantially more resistant—for WT, the D_10_ (the dose at which 10% of irradiated cells maintained the ability to form colonies) was achieved at a dose of appx. 2500 Gray (Gy, or J/kg, a unit describing the dose of absorbed ionizing radiation), while it was greater than 4000 Gy for the evolved strain.

### 3.2. Pressure-Assisted Lysis of E. dermatitidis Cells for Protein Extraction and Shotgun Proteomics

Fungi have rigid cell walls that contain a complex mixture of polysaccharides, proteins, and lipids, rendering them difficult to lyse [44]. Another challenge posed by *E. dermatitidis* is its constitutive production of melanin (Figure S1), which additionally stiffens the cell wall [19] and inhibits the activity of cell wall-degrading enzymes, making the common yeast lysis method of protoplasting less tenable in this organism [20]. Few methods, in general, are present in the literature that describe the efficient lysis of black yeasts. SDS, high pH solutions, and the boiling of samples can all weaken the cell wall and allow for easier lysis [45], but they also may induce chemical changes to molecules of interest and are generally not appealing for use in mass-spectrometry-based shotgun proteomics [46].

We therefore employed an alternative approach in order to develop a simple and consistent method for protein extraction—rupturing the cell wall through physical force [32]. To determine how effectively this type of lysis technique works in extracting proteins for proteomic analysis, we tested two separate methods: Bead-beating and pressure-assisted lysis (see Materials and Methods). For this experiment, three cultures of WT *E. dermatitidis* were grown and split into two equal samples and treated with these methods, prior to protein quantification and analysis with mass spectrometry. We observed that each method consistently produced a subset of extracted proteins, but that processing samples with the OneShot allowed for the identification of more proteins with fewer missing values than processing samples by bead-beating (Figure S2). Therefore, we used this lysis method for all subsequent experiments.

### 3.3. The Proteomic Responses of Two E. dermatitidis Strains to γ-Radiation Exposure

After establishing a lysis method for simple extraction of *E. dermatitidis* proteins (Figure S2), we performed the proteomics experiment characterizing the response of each strain to γ-radiation exposure. Similar to our previous transcriptomics experiments [9,47], a moderate radiation dose (500 Gy) was used in order to avoid complicating the results due to the presence of a high number of dead cells, especially in one strain compared to another. Exposure to this dose results in killing of approximately 40% of *E. dermatitidis* cells in both the WT and WTE strains (Figure 1B). As described in the Materials and Methods, after irradiating cultures, we incubated them in fresh medium for 1 h to allow them to mount a response.

**Figure 2:**
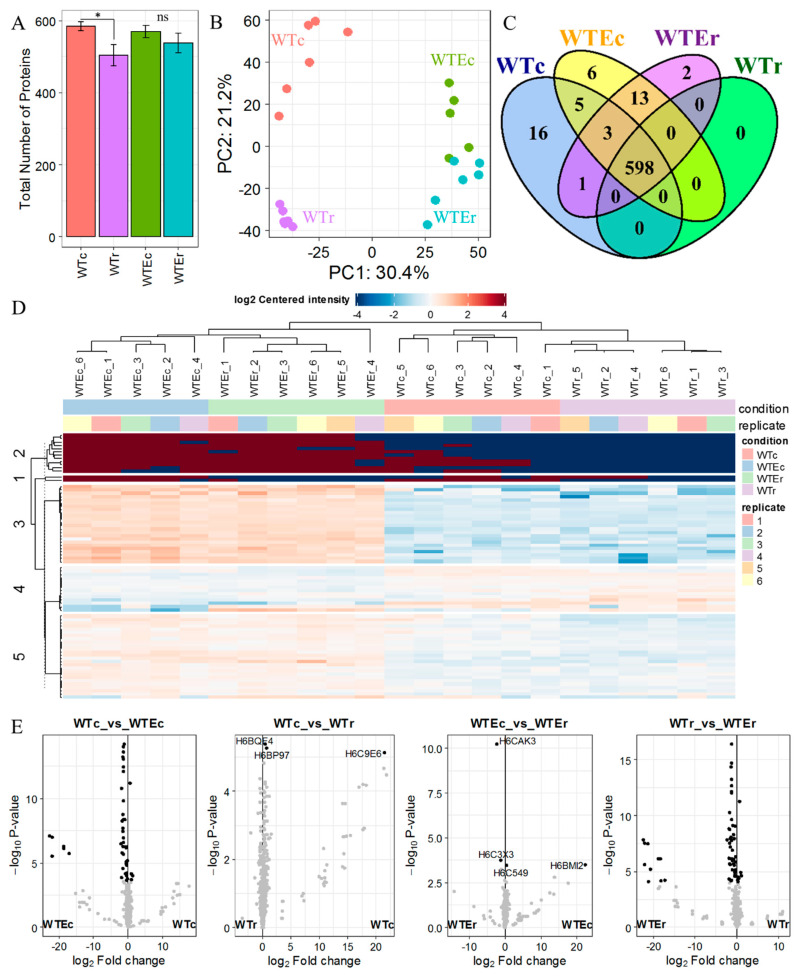
Proteomic response to γ-radiation exposure in WT and Evolved *Exophiala dermatitidis* strains. (**A**) Total number of proteins identified in each condition. * indicates significance (ANOVA, *p* < 1 × 10^−5^). (**B**) PCA of the total proteomic dataset. Each point represents a biological replicate with color corresponding to condition. (**C**) Venn diagram depicting overlap of proteins identified in greater than 4 replicates of each condition (>3 for the Evolved control). (**D**) Heatmap of significantly differentially enriched proteins. Each row represents a single protein and each column represents one replicate, grouped by k-means clustering. Cell colors represent abundance values in comparison to a centered intensity. (**E**) Volcano plots of pairwise comparisons, with –log_10_ unadjusted *p*-values plotted against the log_2_ fold change. Black points represent differentially enriched proteins at α = 0.05 after FDR adjustment. (WTc = WT cultures with no radiation exposure; WTr = WT cultures exposed to radiation; WTEc = evolved WT strain with no radiation exposure; WTEr = evolved WT strain exposed to radiation).

The initial results from this experiment are presented in Figure 2. They allow for several broad conclusions to be made regarding the differences between these strains. First, the average number of proteins identified per sample in the analysis was lower in irradiated samples for both strains, but this decrease was only significant for the WT samples (One way ANOVA, *F*(2,19) = 13.87, *p* = 5 × 10^−5^; Tukey’s HSD, WT vs. WT irradiated *p_adj_* = 5 × 10^−5^, Evolved vs. Evolved irradiated *p_adj_* = 0.09) (Figure 2A).

We can conceive of two possibilities for this consistent decrease in identifiable proteins after irradiation. First, the expression of transcripts encoding these proteins could be downregulated. Indeed, we have observed repression of several hundred genes, including most ribosomal subunits, during γ-radiation recovery [9]. However, the recovery process of 1 h is likely not long enough to see such a dramatic lowering of protein abundance—most of the changes are from proteins that are simply not observed at all in the irradiated samples. Additionally, we have also shown that protein production after irradiation is important for survival, and we see a similar amount of genes being upregulated after irradiation [9]. Therefore, we suggest a second possibility—the decrease is due to either increased protein modification or degradation in the WT strain. Both of these processes are known to occur as a result of γ-radiation exposure, primarily due to both the activity of the proteasome to clear damaged cellular constituents, and the well-known phenomenon of radiation-induced protein oxidation [5,47,48,49,50]. Either of these processes would, in turn, make protein identification using MS-based methods less reliable [51,52,53,54]. In this case, the lower levels of WT protein may reflect the increased sensitivity of these cells and macromolecules inside the cells to γ-radiation compared with the Evolved strain. Oxidation manifests in proteins as carbonylation at many different possible sites that are difficult to identify using shotgun proteomics. However, a principle component analysis of all proteomic samples demonstrated that the WT irradiated samples were strongly clustered together much more than the rest of the samples (Figure 2B). This suggests that exposure to γ-radiation affected the WT strain in a strong and consistent manner, and that this did not occur to nearly the same extent in the Evolved strain.

However, in general, the proteins observed in the datasets exhibited a large overlap among all conditions, with 598/644 of the total proteins identified in the entire experiment being detected in every sample set (Figure 2C), and only a small set of proteins observed exclusively in the Evolved strain samples (*n* = 21/644). Therefore, we performed statistical analysis that incorporated protein intensity information to further characterize the differences between these groups (Table S1, and see Methods), which revealed more distinct differences. Figure 2D shows a heatmap depicting intensity values for the 72 proteins that were found to be present in different levels (with statistical significance) between two or more conditions. As the heatmap demonstrates, the data cluster to a greater extent by the genetic background of the strain rather than by exposure to γ-radiation. This was also reflected in the significantly different proteins identified in pairwise comparisons between the conditions. For example, as shown in the volcano plots in Figure 2E, there are almost no proteins exhibiting statistically significant changes in protein intensity (i.e., the observed amount) between the irradiated and non-irradiated samples for each strain (bold dots, two central plots), while we identified several dozen proteins that fit such criteria when comparing the WT and evolved datasets for each condition (far left and right plots).

**Figure 3:**
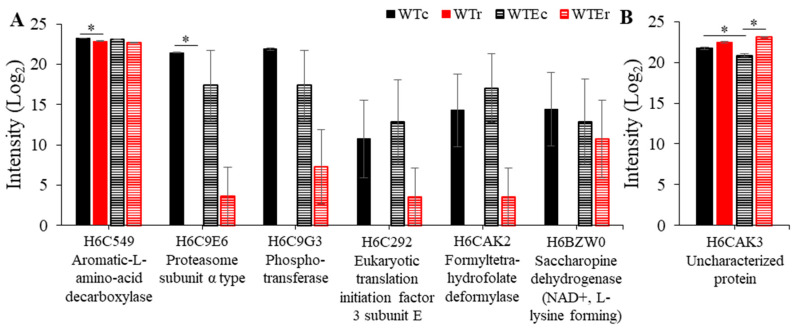
Intensity levels of proteins decreased (**A**) and increased (**B**) after irradiation in both WT and evolved strains. Error bars represent standard error. Asterisks indicate instances of statistically significant comparisons.

Overall, it is apparent that there was not a substantial change in the proteome in response to γ-radiation exposure. In WT, γ-radiation exposure only resulted in a significant change to the intensities of three proteins: The 40S ribosomal protein S9, mannose-1-phosphate guanyltransferase, and an α-subunit of the 20S proteasome, and these were all seen at lower intensities after exposure (Table 1 and Figure 3). Likewise, only four proteins were present at significantly different intensities after irradiation in the Evolved strain: The Aromatic-L-amino-acid decarboxylase and (R,R)-butanediol dehydrogenase decreased in observed amount after irradiation, while an uncharacterized protein, HMPREF1120_008138, as well as an NADH-ubiquinone oxidoreductase 23 kDa subunit, mitochondrial protein were both seen in increased amounts. Notably, even when differences did not reach significance, the direction of changes in protein intensity as observed by MS was the same for each of these proteins in both strain backgrounds (Figure 3).

Another notable observation was that the only protein observed to be present in a relatively higher level (with statistical significance) after irradiation exposure was HMPREF1120_008138, the gene transcript of which was also observed to be strongly induced in the WT strain in response to γ-radiation [9]. These two observations, particularly when considering the small amount of changes we observed otherwise, place this protein as a prime candidate in the *E. dermatitidis* radiation response. However, deleting this gene did not result in increased sensitivity to γ-radiation [9]. In total, then, there were minimal proteomic changes observed after exposure to γ-radiation in either strain, and the proteins that were observed did not suggest the overrepresentation of any specific functional pathways. It has long been uncertain whether the transcriptomic and proteomic changes that occur after γ-radiation exposure or DNA damage in general are important for cell survival [9,25,47,55]. The results we show here provide further evidence that recovery after irradiation is complex and may, at least in part, be mediated by proteins that are present prior to irradiation, or by mechanisms that do not necessarily rely on changes in protein amounts in order to operate.

### 3.4. Proteins Associated with Evolution of Higher Radioresistance

There was a much larger difference between the two strain backgrounds, so we focused our subsequent analyses on this comparison and identified several interesting patterns. Importantly, from the genomic analysis, no genetic variants were identified in or adjacent to genes encoding proteins that we detected in either WTE or WT (Table S2). This meant that few insights could be gleaned from simply analyzing the genomic data, although we did observe at least two genes that could potentially indirectly effect transcription and protein production. First, the gene HMPREF1120_01778 possessed a missense mutation. This gene is predicted to encode a protein with a Homeobox domain, which would suggest that it could regulate gene expression. The other gene is HMPREF1120_00088, which encodes a predicted aminopeptidase. As protein damage and degradation is a known aspect of irradiation, this mutation could lead to changes in protein clearing rates. The effects of the mutations we observed require further exploration, including genetic manipulation, and will be addressed in future studies

**Figure 4:**
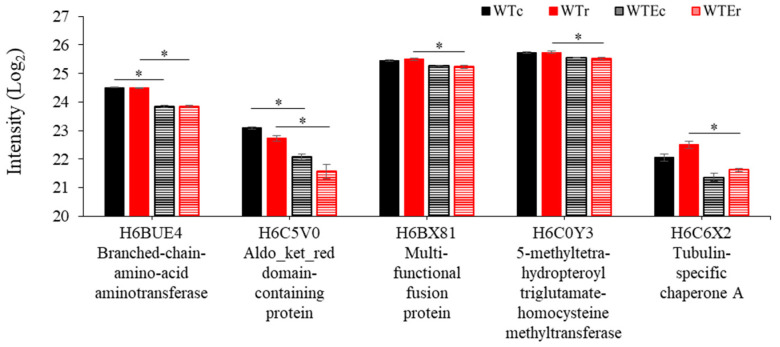
Intensity levels of proteins decreased in the Evolved strains, in comparison with WT strains. Note that only a portion of the *Y* axis is shown to highlight the small, but significant, differences in intensity levels. Error bars represent standard error. Asterisks indicate instances of statistically significant comparisons.

For the proteins that we did identify, most of the differences were from those that were seen in higher relative amounts in the evolved strains. Of the proteins that exhibited statistically significant changes in intensity between the WT and the Evolved strains, 97.9% (*n* = 47/48) and 89.7% (*n* = 52/58) were observed at higher relative intensities in the Evolved strain background under control and irradiated conditions, respectively (Table S2). Thirty-nine of these proteins, moreover, were shared between these two groups. This suggests immediately that increasing production of a certain subset of proteins marks the evolutionary process of this strain, and that this feature is relatively unchanged by exposure to γ-radiation. The remaining proteins, which exhibited lower intensities in Evolved strains, were all involved in amino acid processing, including a branched-chain-amino-acid aminotransferase, which is involved in catabolism of branched chain amino acids (isoleucine, leucine, and valine) [56], an L-glutamate γ-semialdehyde dehydrogenase-domain containing protein, which is involved in the proline degradation pathway and glutamate degradation [57], and a 5-methyltetrahydropteroyltriglutamate-homocysteine methyltransferase, which is involved in methionine synthesis and glutamate catabolism [58] (Figure 4). These suggest a decrease in glutamate and amino acid catabolism, which may be related to changes in the overall cellular redox balance [59].

**Figure 5:**
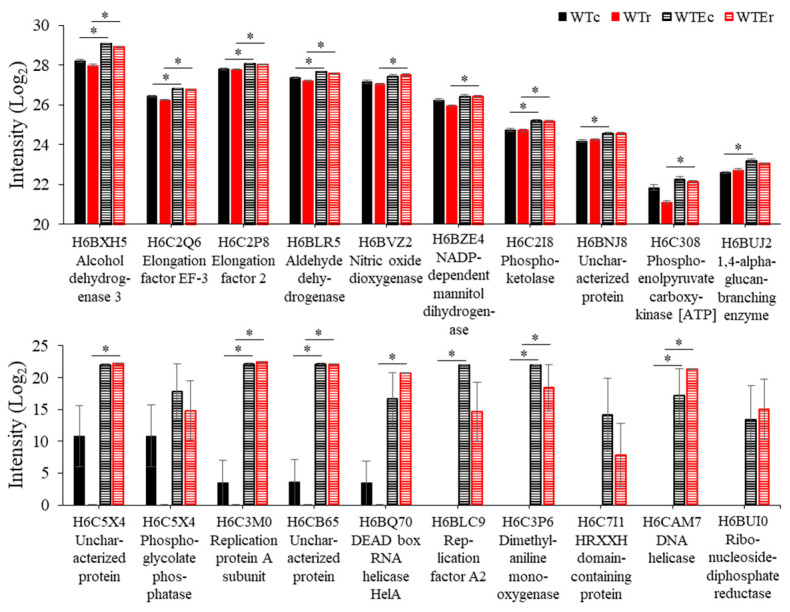
Intensity levels of non-ribosomal proteins increased in the Evolved strains, in comparison with WT strains. Note that only a portion of the *Y* axis is shown in the upper panel to highlight the small, but significant, differences in intensity levels. Error bars represent standard error. Asterisks indicate instances of statistically significant comparisons.

**Figure 6:**
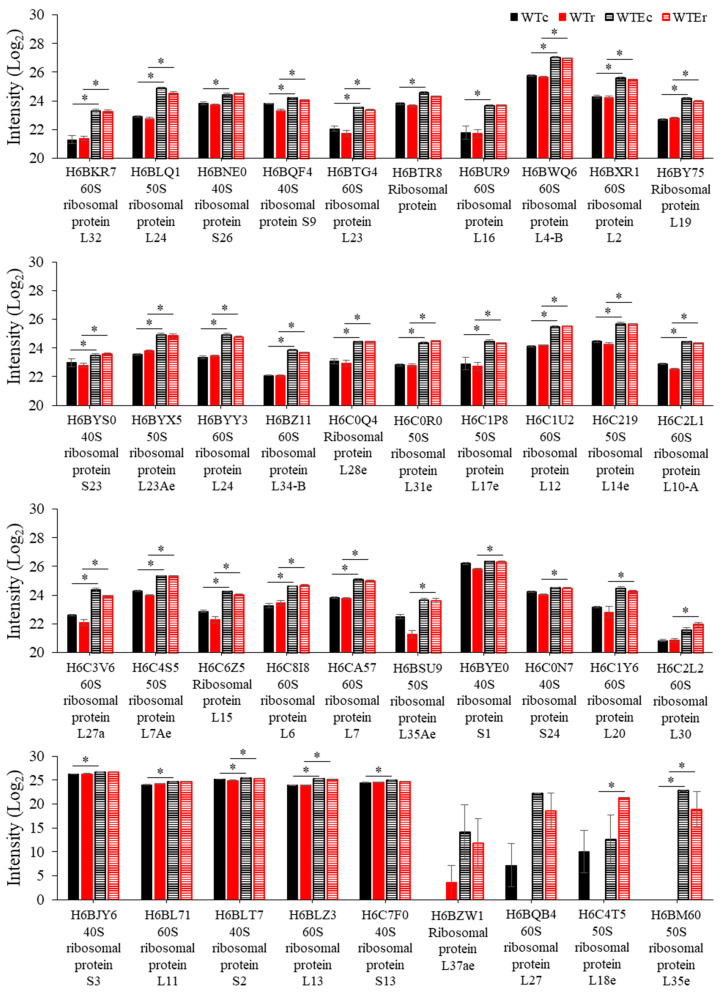
Intensity level readings of ribosomal proteins increased in the Evolved strains, in comparison with WT strains. Note that only a portion of the *Y* axis is shown in the first three panels to highlight the small, but significant, differences in intensity levels. Error bars represent standard error. Asterisks indicate instances of statistically significant comparisons.

A much more distinct pattern was obtained when focusing on the proteins that were increased in the observed relative amount. Figure 5 and Figure 6 show data for this group, and from an analysis of these proteins, several patterns emerge. First, we saw moderate increases in several proteins associated with metabolism and redox balance, including aldehyde and alcohol dehydrogenases, nitric oxide dioxygenase, and an NADP-dependent mannitol dehydrogenases. These changes may indicate again a subtle change in the metabolism of this strain, although we did not observe it. Second, several proteins involved in DNA replication and nucleic acid processing, which we observed to be highly induced after irradiation in our previous transcriptomic studies [9,41], were observed at a much higher intensity in the Evolved strains, including Replication factor A2, a Replication protein A subunit (single-stranded DNA binding during repair and replication), ribonucleoside-diphosphate reductase (deoxyribonucleotide synthesis) and a DNA and an RNA helicase. This is significant due to the role that replication plays in DNA repair, and may suggest that Evolved cells have increased concentrations of limiting DNA repair proteins present during exposure, allowing them to recover more efficiently through better processing of damaged DNA sites during replication or other repair processes [60].

Finally, the remaining proteins that are observed at relatively greater intensities in the Evolved strains were all involved in one process—protein translation. In addition to proteins involved in translation—Elongation factor 2 and Elongation factor EF-3 (Figure 5), a remarkable 28 out of 47 in the control condition (FDR = 1.38 × 10^−32^) and 30/52 (FDR = 1.48 × 10^−34^) in the irradiated condition were components of the ribosome. Gene Ontology Enrichment analysis using FungiFun software [42,43] (see Methods) determined that genes involved in the Biological Process of translation were accordingly enriched to a highly significant extent in both the control condition (FDR < 1.38 × 10^−32^) and the irradiated set (FDR < 1.48 × 10^−34^), while no other categories were enriched with a cutoff of FDR < 0.01. Data representing the comparative intensities of these proteins is in Figure 6. These proteins represent a large portion of the ribosomal proteins present in the *E. dermatitidis* genome (*n* = 122 total), considering the amount of proteins identified in these samples, and their presence here is clear evidence that ribosome production is increased in this Evolved strain.

## 4. Discussion

Some fungal species have been found to be extremely resistant to γ-radiation [9,61,62], which has motivated our efforts to identify fungal-specific mechanisms of γ-radiation protection and recovery. Historically, studies of γ-radiation resistance in most organisms have focused on DNA repair, based on the observation that DNA damage (e.g., single- and double-strand breaks) is the cause of cell death after γ-radiation exposure [2,3,63,64]. However, these studies have not yet uncovered any striking differences in this pathway between organisms of differing radiosensitivity. Similar to the case in bacteria, where the extent of the DNA repair protein repertoire does not appear to correlate to radioresistance [5,65,66], eukaryotes perform DNA repair using a set of proteins that are highly conserved, and fungi in general appear to follow a more simplified process than the more radiosensitive mammals [3,4,64,67,68,69,70]. It is still currently unclear, in fact, whether γ-radiation resistance is the result of more efficient DNA repair enzymes, or if more subtle factors are involved. Addressing this requires analysis of the γ-radiation response in both sensitive and resistant organisms.

We have recently begun these types of comparative analyses in fungi. In the case of *E. dermatitidis*, the yeast used in this study, the vast majority of DNA damage appears to be repaired by homologous recombination, with the canonical recombination protein Rad52 being extremely important [9]. This is also the case for *Saccharomyces cerevisiae* [3,71,72], but not for the more closely-related *Aspergillus niger*, which is more dependent upon non-homologous end-joining [73,74]. Moreover, *E. dermatitidis* (LD_10_ = ~2500 Gy [9]) is far more resistant to γ-radiation (e.g., X-rays and γ-rays) than *A. niger* (LD_10_ = ~400 Gy [73,74]) and the even more closely related *Exophiala lecanii-corni* (LD_10_ = ~250 Gy [9]), while several strains of *S. cerevisiae* have been collected that exhibit a wide range of radiation resistance [10]. These considerations, as well as the conservation of function of most DNA repair enzymes across great evolutionary distance [4,67,72,75,76] suggest that understanding the observed diversity in γ-radiation resistance is less a matter of looking for specific mutations rather than obtaining a deeper understanding of how certain organisms respond to γ-radiation exposure.

One way to do this is through comparative transcriptomics. It is possible that certain resistant organisms mount a more dramatic and effective response when exposed to γ-radiation. Indeed, our group and others have observed robust changes in the transcriptome in both the highly resistant *Cryptococcus neoformans* and *E. dermatitidis* in response to various levels of γ-radiation [9,47,62]. However, the majority of the highly induced genes that were subsequently interrogated were not involved in γ-radiation resistance. A transcriptomic and functional genetic analysis of *Deinococcus radiodurans* produced the same result [65]. Moreover, induction of *RAD52* after irradiation is observed in *S. cerevisiae* [77] and in *E. dermatitidis* [9,41], but the regulation of its expression, or of *RAD54*, do not appear to be required for them to carry out their normal functions [55,78], and the transcriptomic response (e.g., induction of DNA repair proteins, inhibition of growth) is either largely similar across many organisms of varying sensitivity to γ-radiation [66,77,79,80,81], or has been shown not to assist in the identification of DNA repair genes at all [25].

The lack of clarity in these results is central to an ongoing debate in radiation biology, the cause of γ-radiation -induced cell death. Though DNA damage is certainly the proximate cause of cell inactivation, as unrepaired lesions affect genome stability, the ultimate cause appears to be the inability of proteins to repair DNA damage, due to irreversible carbonylation of functional amino acids, caused by the free radicals produced by γ-radiation -induced radiolysis of intracellular water [5,82]. Indeed, one factor that appears to be similar among many γ-radiation -resistant organisms is their comparatively lower level of protein oxidation after γ-radiation exposure [7,10,83,84,85], which allows them to still carry out the processes involved in repair. Taking this into account forces one to view ‘omics-level analyses in a different light: If the maintenance of protein integrity is the primary factor in resistance, then induction of genes is not necessarily important for recovery, which could lead to some of the negative results in genetics experiments that have been informed by gene expression data. Alternatively, other experiments have observed that protein production after irradiation is important for recovery [9,86,87,88,89].

The results we present, on the other hand, lend some clarity to this situation. The first important finding we observed is that consistently less proteins were identified in the WT irradiated sample. Though the difference was small, likely due to the moderate dose that we used (which resulted in almost no difference in survival rate), the effect resulted in strong clustering of the samples from this condition in a principle component analysis, something that was not observed in the evolved strain, leading us to consider that the changes may be induced by the γ-radiation exposure itself. In this case, because the more resistant Evolved strain did not exhibit such a result, this could be due to the increased level of protein oxidation in the WT strain as a result of its relatively higher sensitivity. Oxidation of proteins would change the peptide mass of proteins, rendering their identification by MS more difficult [90]. We plan to explore this effect by looking at levels of overall carbonylation (i.e., protein oxidation) in both strains, performing targeted analysis of post-translational modification of select proteins in both strains after irradiation, and completing the same experiment presented here with larger γ-radiation doses, which we hypothesize would be expected to produce a much larger effect in the WT cells if increased protein oxidation is resulting in this difference in protein identification [90].

The second major result we obtained was that there were few proteins that we observed changing in their relative amounts in response to γ-radiation exposure. We were surprised, because it suggested that the Evolved strain did not possess any obvious, unique inducible response. It was also in stark contrast to the transcriptomic response that we have previously observed—1 h after a dose of 1000 Gy, approximately 4000 genes were differentially expressed (with 2000 induced [9]), while here, six proteins decreased and one protein increased in spectrum intensity after γ-radiation. This suggests to us that there are two things that could be happening within the Evolved strain to assist in its improved response. One is that there is a substantial delay in protein synthesis compared to transcription (i.e., past the 1 h recovery period), so protein production occurs at later time points than our recovery period covered. Because acute ionizing radiation exposure causes extensive oxidative stress, and protein synthesis (including changes in proteomic composition) can occur at time scales much lower than 1 h [9,41,91], we typically consider this length of time to be ample to observe changes at any level that can be scrutinized by current -omic technologies. However, the surprisingly small number of changes that we observed here suggests that a more exhaustive and extended time course should be explored in the future with regard to any proteomic response in this organism, and particularly with the response to γ-radiation exposure, as there is evidence in other fungi of different patterns of regulation over time, upon irradiation [62,77]. Another possibility is that the gene induction we have previously observed is unimportant, at least for these strains, and the factors that are responsible for the improved γ-radiation resistance in the Evolved strain are constitutively present. Finally, the resolution of proteomics, compared with RNA-seq, may be low enough that subtle changes in protein levels are occurring but may not easily be captured by this method. In general, we would pursue only proteins that exhibit large increases in abundance rather than those with minor changes, but in any case, the relatively low level of proteins identified here compared to transcriptomics experiments should be taken into consideration, and protein extraction and identification techniques optimized for this organism in the future.

Taking into account all of the observations we have obtained so far, we believe that a combination of these two things are occurring. This is because of the final observation of this proteomics experiment—the increase in abundance, in the Evolved strains, of two groups of proteins—single-stranded DNA binding proteins and constituents of the ribosome. Single-stranded DNA binding proteins such as the replication factor A complex are essential for most contests where DNA must be unwound, and therefore play a large role in DNA replication and repair [60,92]. The presence of higher levels of proteins in this complex along with a DNA helicase may indicate an augmented ability to unwind and stabilize DNA that needs to be repaired. Exploration of this hypothesis will require further study.

The change in ribosomal proteins was much more prominent. As mentioned above, we previously observed that inhibiting protein synthesis diminishes survival in WT after γ-radiation exposure, which suggests that some factors involved in cell recovery are made after exposure, and that post-irradiation induction of certain genes could still be important. However, if this were the case, many proteins would still need to be present and functioning after irradiation in order for induction to occur, most importantly the ribosomes. We therefore hypothesize that the changes we observed in the intensity of peptides from ribosomal proteins in the Evolved strain used here suggests an overall increase in its cellular ribosome content, which would indicate that this strain has an improved ability to maintain protein synthesis after irradiation.

This hypothesis opens up an intriguing avenue for understanding radiation biology. Ribosomal genes are under strict control based on growth rate and nutrient levels in the environment [93,94]. Generally, when irradiated, ribosomal genes are often seen to be highly repressed compared to non-irradiated samples [9,47,77], a phenomenon that is coupled to cell cycle arrest [95,96]. An abundance of ribosomal DNA gene copies that do not get transcribed, in fact, is important for sister chromatid cohesion, and therefore DNA repair, in budding yeast [97]. If the expression patterns we observed in our transcriptomic experiments [9] reflect an actual decrease in ribosome levels after irradiation, the combined effects of lower gross ribosomal content with which to produce new proteins, along with the increased ribosomal oxidation, could result in ribosomal integrity being a major factor in the recovery of cells from IR. In agreement with this, a decrease in efficiency of ribosomes results in increases in protein oxidation, and specific mutation that blocks the function of ribosomal protein S27-like results in radiation sensitivity in humans [98]. Interestingly, the peroxiredoxin responsible for protecting ribosomes from oxidation in budding yeast, TSA1, is important for resistance to DNA damage [99,100], and its closest homolog was observed to be induced after γ-ray exposure in *E. dermatitidis* [9].

In the Evolved strain, the observation that many ribosomal proteins are present in increased observed intensities suggests that the control over ribosomal synthesis has somehow been relaxed. If this finding is significant to its γ-radiation resistance phenotypes, brief misregulation of ribosomal content could be used to augment recovery in other types of cells, by increasing the cell’s ability to produce proteins involved in recovery. Several follow-up experiments will allow us to address whether this proposal is supported by the evidence, including—(1) determining whether ribosomal biogenesis genes are misregulated in this or other evolved strains, (2) determining whether ribosomes are more or less oxidized after irradiation through targeted analysis of constituent proteins in the evolved strain, (3) testing whether a direct decrease in ribosomes, such as may occur through treatment with rapamycin, sensitizes *E. dermatitidis* to γ-radiation [101] as has been seen in some mammalian cell lines [101,102], (4) measuring the rates of protein synthesis before and after irradiation in Evolved strains compared with WT strains, and (5) identifying the actual transcripts that are translated following irradiation, which has been shown to provide a much better indication of what proteins are being made and are important for the recovery process [103]. Many lines of investigation, therefore, are opened by this data, validating for us the usefulness of proteomics to exposure, possibly to a better extent than comparative transcriptomics, the pathways involved in recovery from γ-radiation exposure, and we recommend this method for exploring aspects of fungal stress biology in the future.

## Figures and Tables

**Table 1:** List of proteins found in significantly different relative amounts after irradiation in WT and Evolved (WTE) strains, along with their predicted functions and the Log_2_ Fold Change (FC) values for the given comparison in both the current proteomic experiment and our prior transcriptomic experiment [9]. NS = not significant. Not observed = protein was not observed in the specified sample so a fold change in relative amount cannot be calculated.

Condition	Gene Name	Annotation	Proteomics FC	Transcriptomics FC
*WTc vs. WTr*	HMPREF1120_02715	40S ribosomal protein S9	−0.47	−1.05
	HMPREF1120_01741	Mannose-1-phosphate guanyltransferase	−0.74	−1.29
	HMPREF1120_08664	Proteasome subunit alpha type	Not observed in WTr	NS
*WTEc vs. WTEr*	HMPREF1120_08138	Uncharacterized Protein	2.28	3.91
	HMPREF1120_06350	NADH-ubiquinone oxidoreductase subunit, mitochondrial	1.22	NS
	HMPREF1120_07744	Aromatic-L-amino-acid decarboxylase	−0.36	NS
	HMPREF1120_00283	(R,R)-butanediol dehydrogenase	Not observed in WTEr	NS

## Supplementary Materials

The following are available online at https://www.mdpi.com/2073-4425/11/10/1128/s1, Supplemental Figure S1. Melanin production by *E. dermatitidis.* Comparison of melanized wild type (WT) strain (left) with a non-melanized mutant (*WdPKS1*) (right) demonstrating the constitutive production of melanin by this organism. Supplemental Figure S2. Effects of bead-beating (BB) and OneShot (OS) lysis on proteomic results. (A) The total number of proteins identified after BB or OS lysis. * Significantly fewer proteins (*p* = 0.036) were identified in the BB samples. (B) Missing value patterns for all proteins where at least one value was missing. The OS samples have many fewer missing proteins than the BB samples. (C) Pearson correlation showing strong correlation between replicates of each condition but weak correlation between BB and OS. (D) Volcano plot showing differentially enriched proteins. 25 proteins were more significantly abundant or consistently detected in the OS condition compared to 4 in the BB condition. Supplemental Table S1. Total data from statistical analysis of the irradiation proteomic experiment. Columns include ID information for each protein identified, the intensity values observed for the specified protein in each sample, FDR calculations for each comparison, and a True/False value demonstrating whether the difference in abundance for the stated comparison was statistically significant for each specified protein. Supplemental Table S2. All identified SNPs and INDELs in the Evolved strain relative to Ed8656 and WT (passaged). Columns include variant location (chromosome and genetic coordinates), mutation type, name of gene harboring variant (for coding region mutations) or name of nearest gene (for intergenic mutations), gene annotation, as well as the specific nucleotide variant observed in each strain.
