# Supplementary material for: Proteomics Reveals Distinct Changes Associated with Increased Gamma Radiation Resistance in the Black Yeast Exophiala dermatitidis
Source: Genes (Basel). 2020 Sep 25;11(10):1128. doi: 10.3390/genes11101128 (PMC7650708; doi:10.3390/genes11101128)
Supplement: Supplementary file 1 [file genes-11-01128-s001.zip › Supplemental Figures and Tables - Discussion of Lysis Methods and Genome Analysis.docx]

*Pressure-assisted lysis of* E. dermatitidis *cells for protein extraction and shotgun proteomics*

Fungi have rigid cell walls that contain a complex mixture of polysaccharides, proteins, and lipids, rendering them difficult to lyse [26]. Another challenge posed by E. dermatitidis is its constitutive production of melanin (**Supplemental Figure 1**), which stiffens the cell wall even more [19] and inhibits the activity of cell wall-degrading enzymes, making the common yeast lysis method of protoplasting less tenable in this organism [20]. Few methods, in general, are present in the literature that describe the efficient lysis of black yeasts. SDS, high pH solutions, and the boiling of samples can all weaken the cell wall and allow for easier lysis [27], but they also may induce chemical changes to molecules of interest and are generally not appealing for use in mass-spectrometry-based shotgun proteomics [28].

We turned, therefore, to an alternative approach in the hopes of developing a simple and consistent method for protein extraction – rupturing the cell wall through physical force [29]. To determine how effectively this type of lysis technique works in extracting proteins for proteomic analysis, we tested two separate methods: bead-beating and pressure-assisted lysis (see Materials and Methods). For this experiment, three cultures of WT E. dermatitidis were grown and split into two equal samples and treated with these methods, prior to protein quantification and analysis with mass spectrometry. We observed that each method consistently produced a subset of extracted proteins, but that processing samples with the OneShot allowed for the identification of more proteins with fewer missing values than processing samples by bead-beating (**Supplemental Figure 2**). Therefore, we used this lysis method for all subsequent experiments.

*Genetic analysis of* E. dermatitidis *evolved strain from whole-genome sequencing*

Our goal in this manuscript is to determine the proteomic changes that co-occur with increased γ-radiation resistance in this organism, rather than the genetic differences induced by the directed evolution process. To be able to fully detail the proteome of both strains, however, we first determined the mutations that were present in the evolved strain through whole genome sequencing (**Supplemental Table 2**). Relative to WT, the genome of WTE was found to harbor a total of 32 variants, including 20 single nucleotide polymorphisms (SNPs) and 12 insertions/deletions (INDELs). Approximately 53% (17/32) of these variants occurred in intergenic regions, and 13% (4/32) were in the untranslated region (UTR). The remaining 34% (11/32) occurred in protein coding sequences, with 16% (5/32) resulting in a substitution or insertion in the amino acid sequence. This included a missense mutation in an aminopeptidase-encoding gene and two neighboring missense mutations in the gene encoding DNA polymerase delta subunit 3. Approximately 9% (3/32) were characterized as highly disruptive variants, which included frameshift and stop codon gain mutations that are likely to lead to truncated or nonfunctional protein products. These variants occurred in genes encoding a transcription factor, a 3-ketoacyl-(acyl-carrier-protein) reductase, and a hypothetical protein. We plan on pursuing a genetic characterization of this and other radioresistant strains in a separate manuscript, so next, we proceeded to protein extraction.
