# Supplementary figures and images for: Proteomics Reveals Distinct Changes Associated with Increased Gamma Radiation Resistance in the Black Yeast Exophiala dermatitidis
Source: Genes (Basel). 2020 Sep 25;11(10):1128. doi: 10.3390/genes11101128 (PMC7650708; doi:10.3390/genes11101128)

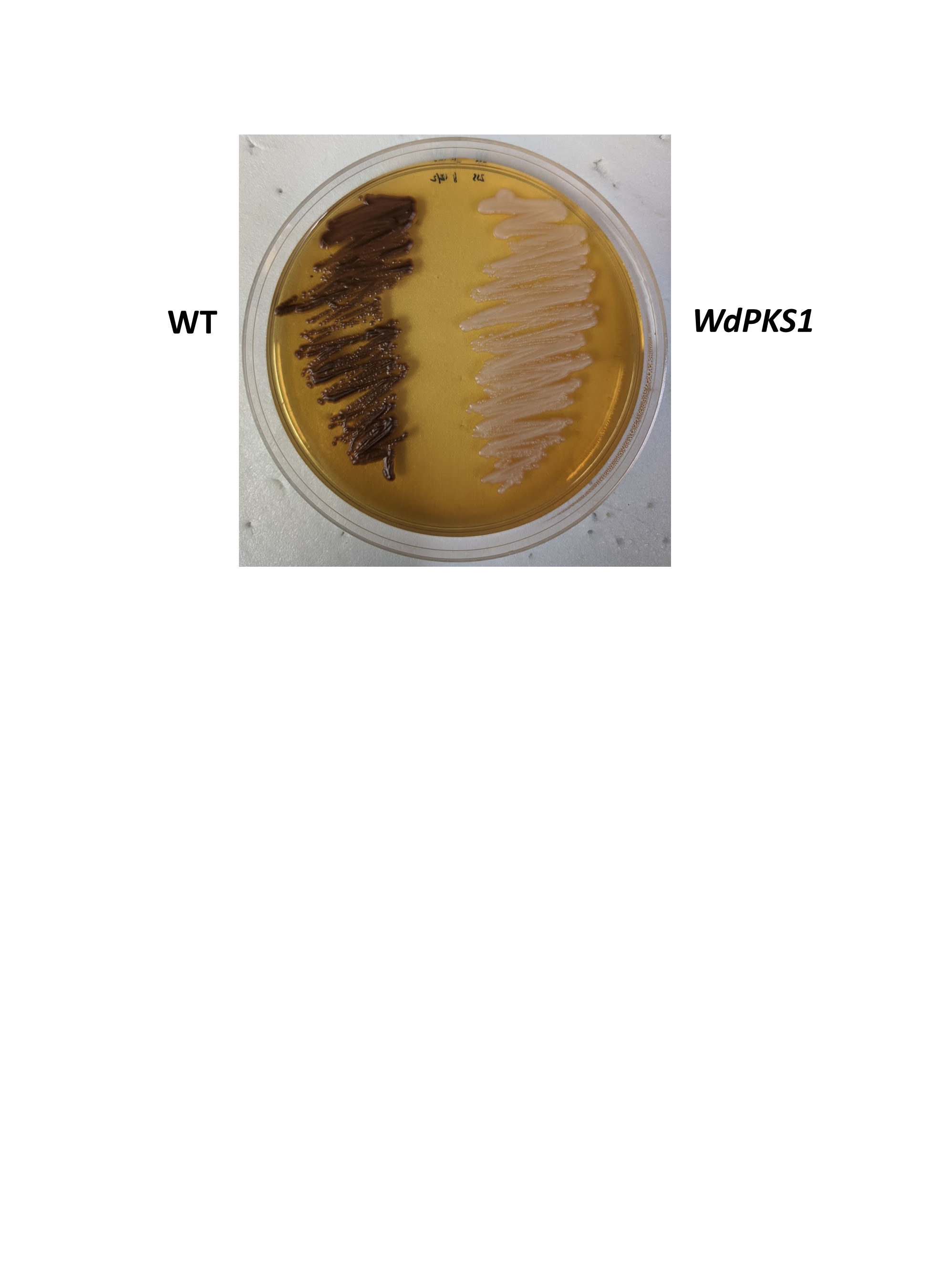

Supplement: Supplementary file 1 [file genes-11-01128-s001.zip › Supplemental Figure 1.tif]

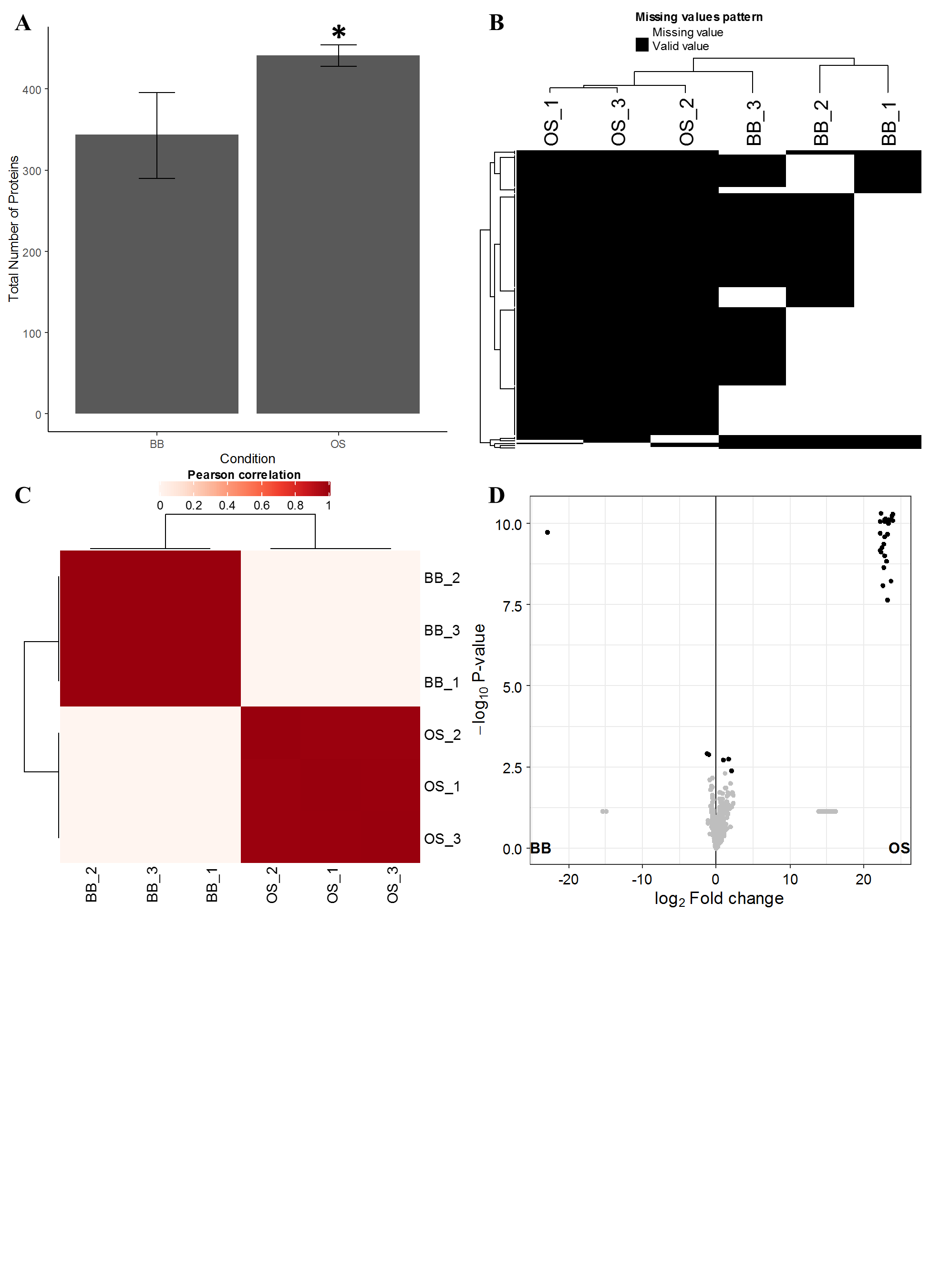

Supplement: Supplementary file 1 [file genes-11-01128-s001.zip › Supplemental Figure 2.tif]
